# Platelets as an indicator of vascular repair in elderly Japanese men

**DOI:** 10.18632/oncotarget.10229

**Published:** 2016-06-22

**Authors:** Yuji Shimizu, Shimpei Sato, Jun Koyamatsu, Hirotomo Yamanashi, Mako Nagayoshi, Koichiro Kadota, Takahiro Maeda

**Affiliations:** ^1^ Department of Community Medicine, Nagasaki University Graduate School of Biomedical Science, Nagasaki, Japan; ^2^ Osaka Center for Cancer and Cardiovascular Disease Prevention, Osaka, Japan; ^3^ Department of Island and Community Medicine, Nagasaki University Graduate School of Biomedical Science, Nagasaki, Japan

**Keywords:** platelets, vascular repair, atherosclerosis, hypertension, CD34-positive cell, elderly men, Gerotarget

## Abstract

Platelets and circulating CD34-positive cells have been reported to contribute to vascular repair (endothelial repair and developing atherosclerosis). And because hypertension is known to be a strong vascular impairment factors, it should also influence the respective numbers of these factors. To clarify the clinical importance of platelets on vascular repair, we conducted a cross-sectional study of 567 Japanese men aged 60-69 who underwent an annual health check-up between 2013 and 2015. Multiple linear regression analysis of non-hypertensive subjects adjusting for classical cardiovascular risk factors showed that although platelet count did not significantly correlate with carotid intima media thickness (β = −0.05, *p* = 0.356), it did positively correlate significantly with the natural log of the number of circulating CD34-positive cells (β = 0.26, *p* < 0.001). In hypertensive subjects, a significant positive correlation was seen between platelets and intima media thickness (β = 0.19, *p* = 0.008), whereas no significant correlation was seen between platelet count and the natural log of the number of circulating CD34-positive cells (β = 0.11, *p* = 0.119). Our results indicate that platelet count is an indicator of vascular repair activity (endothelial repair and developing atherosclerosis). Additionally, hypertension might mask the beneficial effects of circulating CD34-positive cells.

## INTRODUCTION

Recent experimental studies have revealed a correlation between platelets and circulating CD34-positive cells. These studies indicated that platelets promoted the mobilization of bone marrow-derived CD34-positive cells into the peripheral blood [1–8]. Furthermore, platelets are reported to induce differentiation of human CD34-positive cells into endothelial cells [9]. Since CD34-positive cells have been reported to contribute to endothelial repair [10], and platelet rich plasma could enhance the proliferation of bone marrow multi-potent stem cells [6], high levels of platelets in the plasma might have a beneficial effect on endothelial repair. However, platelets are also reported to play an important role in the development of atherosclerotic lesions as an initial actor [11]. A previous study reported that platelets induced the differentiation of human CD34-positive cells into foam cells [9], which are a well-known contributing factor in the development of atherosclerotic lesions. Therefore, high plasma platelet counts may also play a crucial role in the development of atherosclerosis.

On the other hand, hypertension is well-known as a strong endothelial impairment factor [12]. In our previous study, we reported that hypertension masks the beneficial effect of circulating CD34-positive cells as an endothelial repair factor [13]. Therefore, the presence of hypertension might act as a confounding factor in the correlation between platelet count and the number of circulating CD34-positive cells.

To investigate the correlation between platelets and circulating CD34-positive cells as a marker of vascular repair (endothelial repair and atherosclerosis development), we conducted a cross-sectional study of 567 men aged 60-69 who underwent an annual health check-up between 2013 and 2015.

## RESULTS

### Characteristics of the study population

Among 567 elderly men, 238 were diagnosed as hypertensive. Characteristics of the study population by hypertension status are shown in Table 1. In addition to systolic and diastolic blood pressure, hypertensive subjects showed significantly higher BMI and CIMT than non-hypertensive subjects.

**Table 1:** Characteristics of the study population based on hypertension status

	Non-hypertension	Hypertension	*p* value
No. of participants	329	238	
Age, years	65.4 ± 2.5	65.6 ± 2.7	
Systolic blood pressure, mmHg	122 ± 10	150 ± 12	<0.001
Diastolic blood pressure, mmHg	74 ± 8	90 ± 9	<0.001
Body mass index (BMI), kg/m^2^	23.0 ± 2.9	23.9 ± 2.9	<0.001
Serum HDL-cholesterol (HDL), mg/dL	57 ± 14	57 ± 15	0.581
Serum triglycerides (TG), mg/dL	116 ± 80	121 ± 101	0.486
Hemoglobin A1c (HbA1c), %	5.7 ± 0.6	5.7 ± 0.7	0.596
Serum aspartate aminotransferase (AST), IU/L	24 ± 9	26 ± 9	0.083
Serum γ-glutamyltranspeptidase (γ-GTP), IU/L	48 ± 67	52 ± 54	0.446
Serum uric acid (UA), mg/dL	5.9 ± 1.2	5.9 ± 1.2	0.574
Serum creatinine, mg/dL	0.88 ± 0.60	0.84 ± 0.15	0.327
Glomerular filtration rate (GFR), mL/min/1.73m^2^	73.0 ± 15.3	73.2 ± 13.7	0.877
Mean carotid intima-media thickness (CIMT), mm^2^	0.67 ± 0.10	0.70 ± 0.12	0.005
Circulating CD34-positive cells, cells/μL	1.29 ± 1.19	1.36 ± 1.54	0.516
White blood cells (WBC), cells/μL	5538 ± 1388	5655 ± 1432	0.328
Platelets (Plt), ×10^4^/μL	21.7 ± 5.2	22.3 ± 5.1	0.204

values: mean ± standard deviation. p values are age-adjusted value.

### Platelets and other variables in relation to hypertension

From simple correlation analysis (simple correlation coefficient), for subjects without hypertension, platelets showed a significant positive correlation with circulating CD34-positive cells and an inverse correlation with AST, but not with CIMT; and for subjects with hypertension, platelets showed a significant positive correlation with CIMT (Table 2).

**Table 2:** Simple correlation coefficient of platelets and other variables

	Platelets			
	Non-hypertension		Hypertension	
	*r*	*p*	*r*	*p*
No. of participants	329		238	
Age	−0.11	0.053	−0.08	0.237
Systolic blood pressure	0.001	0.982	0.03	0.685
Diastolic blood pressure	−0.03	0.549	0.003	0.959
Body mass index (BMI)	−0.04	0.526	−0.06	0.331
Serum HDL-cholesterol (HDL)	−0.02	0.673	−0.05	0.412
Serum triglycerides (TG)	0.05	0.392	0.07	0.251
Hemoglobin A1c (HbA1c)	0.002	0.978	0.09	0.148
Serum aspartate aminotransferase (AST)	−0.22	<0.001	−0.13	0.054
Serum γ-glutamyltranspeptidase (γ-GTP)	−0.003	0.961	−0.11	0.100
Serum uric acid (UA)	0.04	0.460	−0.06	0.326
Glomerular filtration rate (GFR)	0.09	0.086	0.07	0.313
Circulating CD34-positive cells	0.25	<0.001	0.12	0.075
Mean carotid intima-media thickness (CIMT)	−0.05	0.388	0.17	0.010

r: Correlation coefficient. Circulating CD34-positive cells, TG, and γ-GTP are calculated in logarithm values.

### Platelets and circulating CD34-positive cells, mean CIMT in relation to hypertension

**Figure 1:**
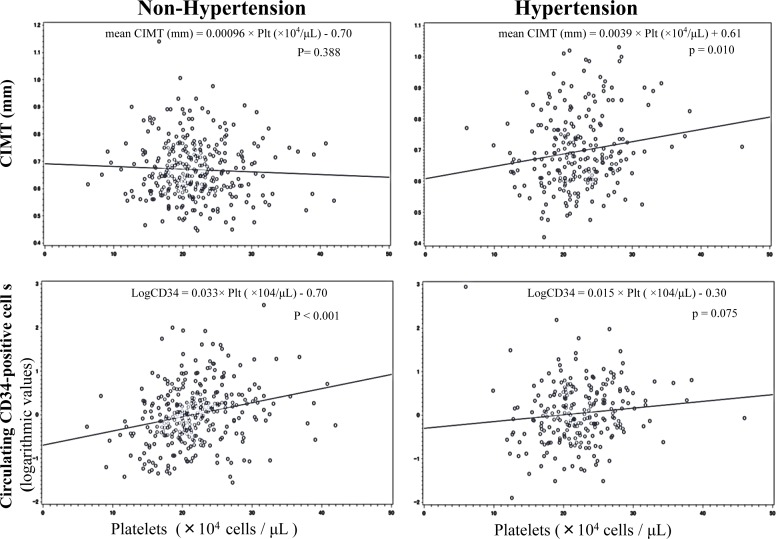
Simple linear regression analyses of platelets and mean CIMT, and circulating CD34-positive cells

From simple linear regression analysis, for subjects without hypertension, platelets showed a significant positive correlation with circulating CD34-positive cells, whereas for subjects with hypertension, no significant correlation was observed. We also found that even though there was no significant correlation between platelets and CIMT in non-hypertensive subjects, a significant positive correlation was seen in hypertension men (Figure 1).

These correlations remained unchanged even after further adjustment for known cardiovascular risk factors (Tables 3, 4).

**Table 3:** Multiple linear regression analysis of mean CIMT with relevant factors adjusted for confounding factors

	mean carotid intima-media thickness (CIMT)					
	Non-Hypertension			Hypertension		
	β	95%CI	*p*	β	95%CI	*p*
No. of participants	329			238		
Age	0.14	(0.03, 0,25)	0.010	0.20	(0.06, 0.33)	0.004
Systolic blood pressure	0.29	(0.05, 0.52)	0.017	0.10	(−0.11, 0.32)	0.347
Diastolic blood pressure	−0.10	(−0.30, 0.10)	0.337	−0.09	(−0.27, 0.09)	0.317
Body mass index (BMI)	−0.01	(−0.13, 0.10)	0.819	0.03	(−0.12, 0.18)	0.688
Serum HDL-cholesterol (HDL)	−0.14	(−0.26, −0.01)	0.032	−0.02	(−0.18, 0.13)	0.752
Serum triglycerides (TG)	−0.15	(−0.28, −0.02)	0.022	0.01	(−0.15, 0.17)	0.927
Hemoglobin A1c (HbA1c)	0.05	(−0.06, 0.17)	0.346	0.03	(−0.10, 0.16)	0.606
Serum aspartate aminotransferase (AST)	−0.07	(−0.19, 0.04)	0.217	0.07	(−0.09, 0.22)	0.389
Serum γ-glutamyltranspeptidase (γ-GTP)	0.09	(−0.03, 0.22)	0.151	−0.10	(−0.27, 0.07)	0.267
Serum uric acid (UA)	0.07	(−0.04, 0.17)	0.230	−0.09	(−0.13, 0.06)	0.232
Glomerular Filtration Rate (GFR)	0.01	(−0.09, 0.11)	0.839	−0.07	(−0.23, 0.08)	0.352
Platelets (Plt)	−0.05	(−0.15, 0.05)	0.356	0.19	(0.05, 0.33)	0.008

β: Standardized parameter estimate. TG and γ-GTP are calculated in logarithm values.

**Table 4:** Multiple Linear Regression Analysis of Circulating CD34-positive cells with Relevant Factors adjusted for Confounding Factors

	Circulating CD34-positive cells					
	Non-hypertension			Hypertension		
	β	95%CI	*p*	β	95%CI	*P*
No. of participants	329			238		
Age	−0.08	(−0.19, 0.02)	0.129	−0.03	(−0.16, 0.10)	0.620
Systolic blood pressure	−0.02	(−0.26, 0.21)	0.857	−0.02	(−0.22, 0.19)	0.872
Diastolic blood pressure	0.06	(−0.14, 0.26)	0.567	−0.01	(−0.19, −0.16)	0.867
Body mass index (BMI)	0.20	(0.08, 0.31)	0.001	0.16	(0.02, 0.30)	0.025
Serum HDL-cholesterol (HDL)	−0.04	(−0.16, 0.09)	0.563	0.02	(−0.13, 0.17)	0.817
Serum triglycerides (TG)	0.19	(0.06, 0.32)	0.004	0.01	(−0.15, 0.16)	0.933
Hemoglobin A1c (HbA1c)	0.05	(−0.06, 0.16)	0.389	0.02	(−0.10, 0.15)	0.733
Serum aspartate aminotransferase (AST)	0.06	(−0.06, 0.18)	0.342	−0.15	(−0.19, −0.00002)	0.050
Serum γ-glutamyltranspeptidase (γ-GTP)	−0.02	(−0.14, 0.11)	0.800	−0.06	(−022, 0.11)	0.509
Serum uric acid (UA)	−0.01	(−0.12, 0.09)	0.802	0.07	(−0.06, 0.21)	0.291
Glomerular Filtration Rate (GFR)	−0.06	(−0.16, 0.04)	0.271	−0.02	(−0.17, 012)	0.741
Platelets (Plt)	0.26	(0.15, 0.36)	<0.001	0.11	(−0.03, 0.24)	0.119

β: Standardized parameter estimate. Circulating CD34-positive cells, TG, and γ-GTP are calculated in logarithm values.

## DISCUSSION

The main findings of present study showed platelet count to be significantly positively correlated with circulating CD34-postive cells, but not with CIMT in non-hypertensive men, and not significantly correlated with circulating CD34-positive cells, but significantly positively correlated with CIMT in hypertensive men. These results demonstrate that platelet count is an indicator of vascular repair activity, and that hypertension might mask the beneficial effects of circulating CD34-positive cells.

This is the first epidemiological study clarifying the correlation between platelet count and the number of circulating CD34-positive cells and CIMT. It is also the first study to clarify the influence of hypertension on these correlations. Even though platelet count indicates endothelial repair activity, a high platelet count contributes to high CIMT in hypertensive men. These findings might indicate an efficient tool to clarify the mechanism of vascular repair in elderly men.

Platelets play an important role in vascular inflammation and vessel wall remodeling [14,15]. Injury to the arterial endothelial cells causes exposure of sub-endothelial components, notably collagen [16,17] and von Willebrand factor [18], resulting in the adherence of platelets to damaged vessel walls and their subsequent activation. Activated platelets (P-selectin-positive platelets) express or release stromal cell-derived factor 1α (SDF-1α) [1], leading to mobilization of bone marrow-derived CD34-positive cells into the peripheral blood in human acute coronary syndrome [2]. Furthermore, platelets secrete vascular endothelial growth factor (VEGF) and sphingosine-1-phosphate (S1P) [3] upon their activation. A previous study also reported that myocardial necrosis with simultaneous elevation of VEGF and SDF-1α caused significant CD34-positive cell elevation in patients with cardiovascular disease [4]. Additionally, plasma levels of S1P have been shown to lead to the trafficking of stem cells from the bone marrow to the peripheral blood [5]. Platelets are a rich source of VEGF, SDF-1α, and S1P [3], and platelet-rich plasma could enhance the proliferation of bone marrow mesenchymal stem cells, which are known to be multi-potent stem cells [6]. On the other hand, stem cells are also known to differentiate into megakaryocytes and to subsequently produce platelets [7]. Therefore, platelet count might positively correlate with the number of circulating CD34-positive cells.

Moreover, CD34-positive cells have been reported to contribute to endothelial repair [10]. Since platelets induce differentiation of human CD34-positive cells into endothelial cells [9], and SDF-1α promotes differentiation of CD34-positive cells to endothelial progenitor cells [1], the positive correlation between platelets and circulating CD34-positive cells might indicate endothelial repair activity.

However, in subjects with hypertension, we found no significant correlation between platelets and circulating CD34-positive cells. Furthermore, in subjects with hypertension, a significant positive correlation was seen between platelets and CIMT.

Since hypertension is a well-known endothelial impairment factor [12], the necessity of endothelial repair in hypertensive subjects is much higher than in subjects without hypertension. Therefore, hypertension has been reported to increase the number of activated platelets in peripheral blood [19]. However, in hypertensive subjects, maximum CD34-positive cell productivity by the bone marrow might easily be attained, followed by a consumptive reduction in circulating CD34-positive cells. Since CD34-positive cells are immature cells, aggressive endothelial repair might cause consumptive reduction in circulating CD34-positive cells because many of them become mature cells (CD34-negative cells) such as endothelial cells, mural cells, and foam cells. Even though the presence of CD34-positive cells in human atherosclerotic lesions was previously observed [20,21], that fact that increased circulating CD34-positive cell count was associated with a decrease in the extent of subclinical atherosclerosis in asymptomatic men [22] might be explained by the influence of this consumptive reduction. Under such conditions, the positive correlation between platelet count and number of circulating CD34-positive cells disappears.

**Figure 2:**
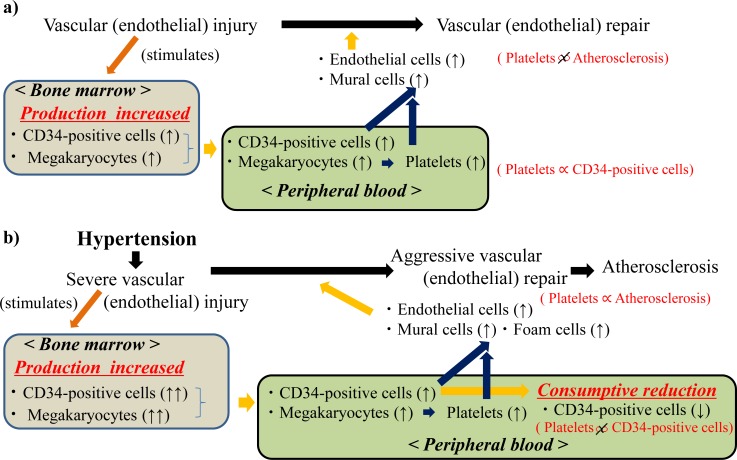
Possible mechanisms underlying the platelet and CD34-positive cell on vascular repair among **a**) subjects without hypertension and **b**) subjects with hypertension

On the other hand, platelets are reported to play an important role in the development of atherosclerotic lesions as an initial actor [11]. Additionally, platelets not only induce differentiation of human CD34-positive cells into endothelial cells, but also into foam cells, which are a contributing factor in the development of atherosclerosis [9]. The existence of circulating CD34-positive cells in hypertensive men with high plasma platelet levels might no longer reduce the risk of atherosclerosis despite CD34-positive cells playing important role in endothelial repair [10]. Therefore, in our study, the significant positive correlation between platelets and mean CIMT is limited to subjects with hypertension. The possible mechanisms underlying the correlation between platelet and CD34-positive cell on vascular repair are summarized in Figure 2. The number of circulating CD34-positive cell is determined by production and consuming.

Potential limitations of this study warrant consideration. Since we have no data regarding activated platelets (P-selectin-positive platelets), the exact influence of active platelets on the circulating CD34-positive cells is unknown. However, a previous study reported that platelet rich plasma enhances the proliferation of bone marrow multi-potent stem cells [6]. Therefore, we believe that the number of platelets correlates with the number of active platelets. Further studies that include data on active platelets are necessary. Additionally, as this was a cross-sectional study, we were not able to establish causal relationships.

In conclusion, our findings demonstrate that platelet count indicates vascular repair activity, and that hypertension might mask the beneficial effects of circulating CD34-positive cells.

## MATERIALS AND METHODS

### Subjects and methods

This study was approved by the Ethics Committee for Human Use of Nagasaki University (project registration number 14051404). Written consent forms were available in Japanese to ensure comprehensive understanding of the study objectives, and informed consent was provided by the participants. The study population comprised 617 male residents aged 60-69 years from the western rural communities of the Goto city and Saza town, who undertook an annual medical check-up from 2013 to 2015 as recommended by the Japanese government.

To avoid the influence of inflammatory disease and hematological disease, subjects with high and low white blood cell counts (≥10,000 cells/μL (*n* = 8) and 1,000 cells/μL< (*n* = 2), respectively) were excluded. Additionally, to avoid the influence of bone marrow- activating medications, subjects taking medicines for anemia (*n* = 3) were also excluded from the analysis, as were persons with missing data (*n* = 37). The remaining patients, comprising 567 men with a mean age of 65.5 years (standard deviation (SD): 2.6; range: 60-69), were enrolled in the study.

### Data collection and laboratory measurements

Body weight and height were measured with an automatic body composition analyzer (BF-220; Tanita, Tokyo, Japan) and body mass index (BMI; kg/m^2^) was calculated. Systolic and diastolic blood pressure was recorded at rest. Hypertension was diagnosed as a systolic blood pressure ≥ 140mmHg and/or a diastolic blood pressure ≥ 90mmHg.

Fasting blood samples were collected in an EDTA-2K tube, a heparin sodium tube and a siliconized tube. The levels of platelets (Plt) and white blood cells (WBC) in samples from the EDTA-2K tube were measured at SRL, Inc. (Tokyo, Japan) using flow cytometry.

Fresh samples (within 24 hours of collection) from a heparin sodium tube were used to determine the number of CD34-positive cells. BD (Beckton Dickinson Biosciences) Trucount^TM^ technology, an accurate and reproducible single platform assay conforming to the International Society of Hematotherapy and Graft Engineering (ISHAGE) guidelines [23, 24] and supported by automated software on the BD FACSCanto^TM^ II system, was used to measure circulating CD34-positive cells. Serum samples were separated to measure the concentration of aspartate aminotransferase (AST) and γ-glutamyltranspeptidase (γ-GTP) using the Japanese Society of Clinical Chemistry (JSCC) standardization method. Triglycerides (TG), uric acid (UA) and creatinine were measured enzymatically. HDL-cholesterol (HDL) was measured using the direct method, while hemoglobin A1c (HbA1c) was measured using the latex coagulation method. Glomerular filtration rate (GFR) was estimated by means of an established method using three variations that had recently been proposed by the working group of the Japanese Chronic Kidney Disease initiative [25]. According to this adapted version, GFR (mL/min/1.73 m^2^) = 194 × (serum creatinine (enzyme method))^−1.094^× (age)^−0.287^.

Measurement of carotid intima media thickness (CIMT) was determined by ultrasonography of the left and right carotid arteries by an experienced vascular technician using a LOGIQ Book XP with a 10-MHz transducer, GE Healthcare, Milwaukee, WI, USA). Mean values for the left and right CIMT were calculated using automated digital edge-detection software (Intimascope; MediaCross, Tokyo, Japan) and a protocol that has been described in detail elsewhere [26].

### Statistical analysis

Distribution of circulating CD34 cells, plasma platelet concentration, and mean CIMT were analyzed. Clinical characteristics of hypertensive and non-hypertensive subjects were compared. Simple correlation analysis (correlation coefficient) of circulating CD34-positive cells, platelets and other variables stratified by hypertension status were performed. In order to determine the correlation between the number of circulating CD34-positive cells and platelets, simple and multiple linear regression analysis accounting for hypertension status was performed, along with simple and multiple linear regression analysis of the mean CIMT and other variables.

Adjustments were made for classical cardiovascular risk factors such as age, systolic blood pressure (mmHg), diastolic blood pressure (mmHg), body mass index, HbA1c (%), HDL (mg/dL), TG(mg/dL), AST (IU/L), γ-GTP(IU/L), UA(mg/dL), and GFR (mL/min/1.73m^2^). Because CD34-positive cells, TG, and γ-GTP had a skewed distribution, logarithmic transformation was performed for simple and partial correlation analysis, and linear regression analysis.

All statistical analyses were performed with the SAS system for Windows (version 9.3; SAS Inc., Cary, NC). Probability values less than 0.05 were considered to be statistically significant.
